# (Pro)renin receptor is involved in mesangial fibrosis and matrix expansion

**DOI:** 10.1038/s41598-017-18314-w

**Published:** 2018-01-08

**Authors:** Kaori Narumi, Emiko Sato, Takuo Hirose, Tae Yamamoto, Takashi Nakamichi, Mariko Miyazaki, Hiroshi Sato, Sadayoshi Ito

**Affiliations:** 1 Division of Nephrology, Endocrinology and Vascular Medicine, Tohoku University Graduate School of Medicine, 1-1 Seiryo-machi, Aoba-ku, Sendai 980-8574 Japan; 2 Division of Clinical Pharmacology and Therapeutics, Tohoku University Graduate School of Pharmaceutical Science, 6-3 Aoba, Aramaki, Aoba-ku, Sendai 980-8579 Japan

## Abstract

(Pro)renin receptor [(P)RR] is expressed in the kidney and is involved in renal injury. Although (P)RR is activated by indoxyl sulfate (IS) and may be related to renal injury, the details remain unclear. We used mouse mesangial cell line SV40 MES13 to investigate the association of (P)RR with mesangial fibrosis or expansion. Furthermore, we examined the correlation between serum soluble (P)RR [s(P)RR] and various laboratory data including serum IS, a uremic toxin that induces renal fibrosis through (P)RR, and pathological indices in chronic kidney disease and particularly in IgA nephropathy patients. *In vitro* study using SV40 MES13 cells revealed that (P)RR expression significantly increased in the presence of IS. IS stimulated the fibrotic factors’ expression, which was significantly suppressed by (P)RR knockdown. Moreover, it significantly increased the expression of matrix metalloproteinase 9 and tissue inhibitor of metalloproteinase 1 via the ERK1/2 pathway. In addition, the s(P)RR level significantly correlated with serum IS and mesangial injury markers in our patients. Our results suggest that (P)RR is associated with mesangial fibrosis and matrix expansion through the IS-(P)RR-ERK1/2 pathway. Clinically, s(P)RR may be a biomarker of mesangial fibrosis and matrix expansion.

## Introduction

(Pro)renin receptor [(P)RR] was initially identified as a member of the renin angiotensin system (RAS) in 2002^1^. There are three forms of (P)RR. Full length (P)RR is cleaved by furin to a single transmembrane form and a soluble form [s(P)RR], which exists in the blood^2^ and urine^3^. (P)RR is ubiquitasly expressed in the kidney including mesangial cells, podocytes and tubular cells^1,4,5^. Binding of renin or prorenin to (P)RR leads to activation of the intracellular signalling of extracellular signal-regulated kinase (ERK) 1/2. This activation is involved in renal damage by releasing transforming growth factor (TGF)-β1 and cytokines. We have demonstrated that (P)RR is expressed in human peripheral blood mononuclear cells (PBMCs), and that renin stimulates ERK1/2 phosphorylation and interleukin-6 release from PBMCs isolated from healthy subjects. We have also shown that (P)RR is present on infiltrating lymphocytes and macrophages around glomeruli in anti-neutrophil cytoplasmic antibody (ANCA)-associated GN^6^. Previous studies have revealed that (P)RR blockade improved glomerulosclerosis in human (P)RR-transgenic rats^7^ and that the serum levels of s(P)RR correlated with the degree of renal dysfunction in chronic kidney disease (CKD) patients^8^. These results suggest that (P)RR is associated with the progress of renal injury.

CKD is defined as a decrease in glomerular filtration rate and persistent proteinuria. Several uremic toxins accumulate in the blood with CKD progress. Indoxyl sulfate (IS), which is a representative uremic toxin and a metabolite of tryptophan derived from dietary protein, also accumulates in the circulation of CKD patients, and induces glomerulosclerosis^9,10^, mesangial cell proliferation^11^ and expression of fibrotic genes such as *TGF-β1* and *type-I collagen* ^12^ in the kidney. In addition, IS promotes cell proliferation and tissue factor expression in vascular smooth muscle cells^13^, and induces *TGF-β1* and *α-smooth muscle actin* expression in proximal tubular cells through (P)RR^14^.

Based on these results, we hypothesized that IS accumulated by renal failure activates (P)RR and contributes to mesangial injury, and that (P)RR could be a marker for evaluating pathological change of CKD. To verify this hypothesis, we examined the effect of IS and other uremic toxins on the proliferation of mesangial cells via activated (P)RR. Additionally, we investigated the relationship between (P)RR and IS in CKD patients.

## Results

### IS enhances (P)RR expression in SV40 MES13 cells

**Figure 1:**
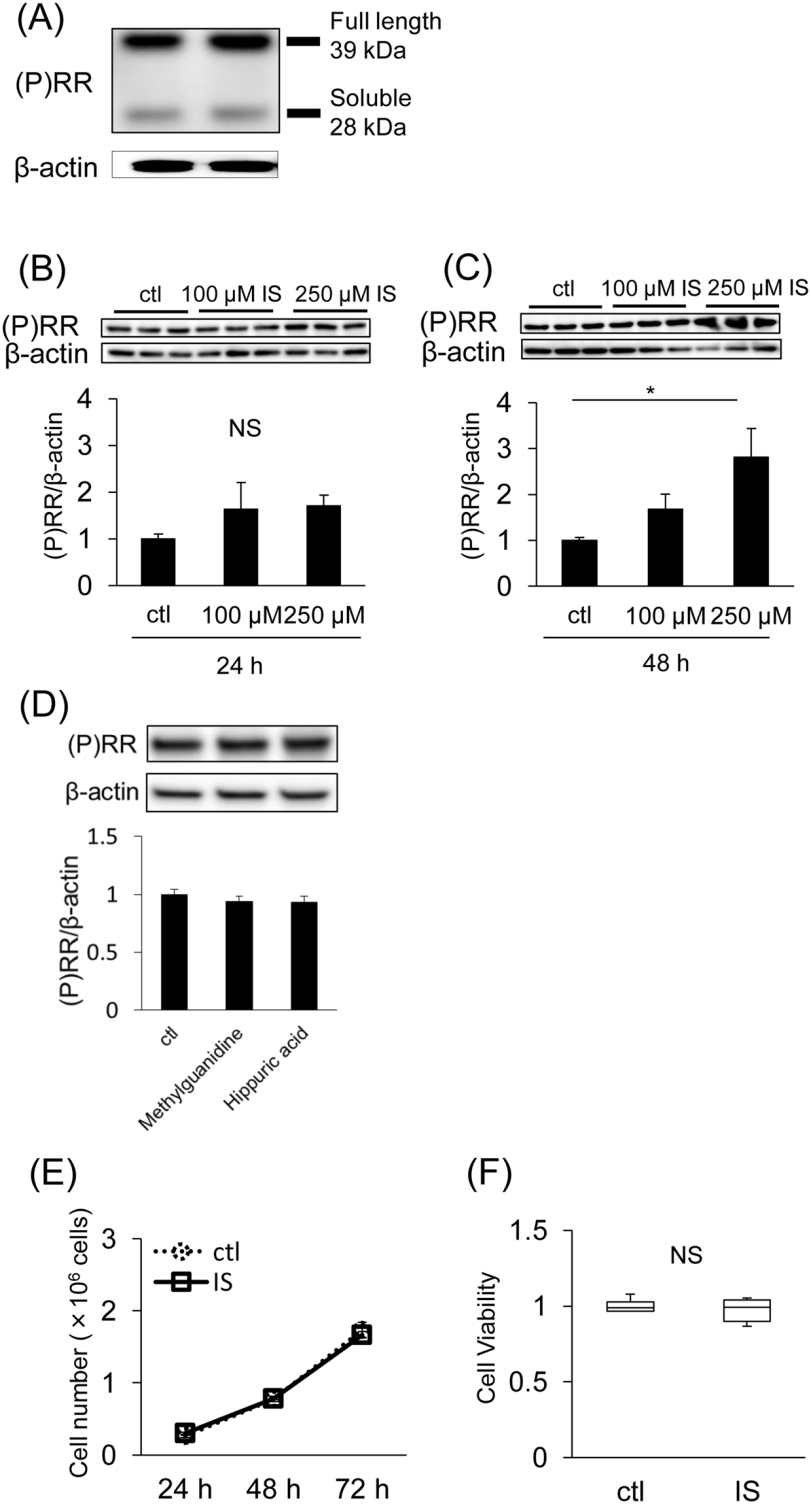
(**A**) (P)RR protein expression in SV40 MES13 cells assessed by western blot analysis. (**B** and **C**) SV40 MES13 cells were incubated with the indicated concentration of IS for 24 h (**B**) or 48 h (**C**). Mean ± SE (n = 6). ctl, control. *P < 0.05, by the Tukey–Kramer test. (**C**) SV40 MES13 cells were incubated with other uremic toxins for 48 h. Mean ± SE (n = 4). ctl, control. (**E**) Cell number after 250 µM IS stimulation (solid line) or 50 mM Tris-HCl incubation [control (ctl); dotted line] for the indicated periods. Mean ± SE (n = 6). (**F**) Cell viability of SV40 MES13 cells incubated with 250 µM IS assessed by the 3-(4,5-di-methylthiazol-2-yl)-2,5-diphenyltetrazolium bromide (MTT) assay (n = 6). Values are presented as the median (central line), interquartile range (box) and range (bars). NS, not significant, vs. control (ctl) by the Student’s *t*-test.

A 39-kDa band corresponding to the full-length (P)RR and a 28-kDa band corresponding to the s(P)RR were detected in SV40 MES13 cells by western blotting (Fig. 1A). Although the (P)RR protein expression did not change after 24 h of IS stimulation (Fig. 1B), it increased by 48 h of IS stimulation (Fig. 1C). However, (P)RR expression did not increase by 48 h of other uremic toxins including methylguanidine and hippuric acid (Fig. 1D). We used 250 µM IS^9^, 3 µM methylguanidine^15^ and 600 µM hippuric acid^15^ in this study because this concentration is equivalent to its mean serum level in patients on hemodialysis. This IS concentration did not affect cell growth or viability (Fig. 1E and F).

**Figure 2:**
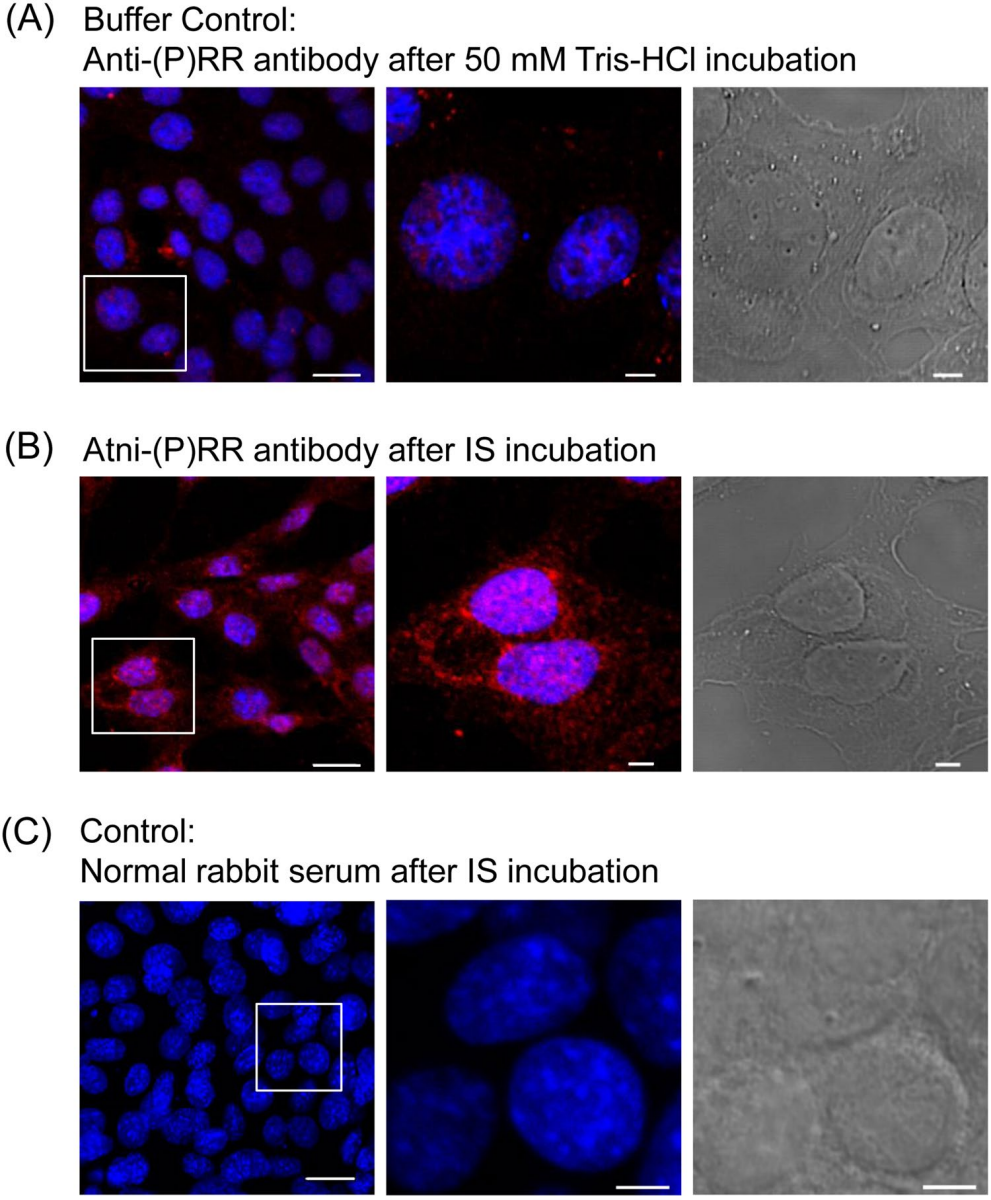
Immunofluorescence of (P)RR in SV40 MES13 cells. SV40 MES13 cells were stained with a (P)RR-specific antibody (red) and the nuclear stain, 4,5-diamidino-2-phenylindole (DAPI, blue). Left: low-power field, Middle: high-power field, Right: bright field. (**A**) Cells after 50 mM Tris-HCl stimulation for 48 h as a control. (**B**) Cells after 250 µM IS stimulation for 48 h. (**C**) Negative control. Scale bars, 20 µm (low-power field) and 5 µm (high-power field and bright field).

Next, we examined the effect of IS on (P)RR expression in SV40 MES13 cells by immunofluorescence analysis. Positive staining of (P)RR was observed after 50 mM Tris-HCl exposure (Fig. 2A). This was the buffer control indicated the baseline levels of (P)RR. The expression of (P)RR was enhanced in the cytoplasm of SV40 MES13 cells after IS exposure (Fig. 2B). No staining was observed in the absence of the primary antibody (Fig. 2C).

### IS enhances the expression of fibrotic genes through (P)RR

**Figure 3:**
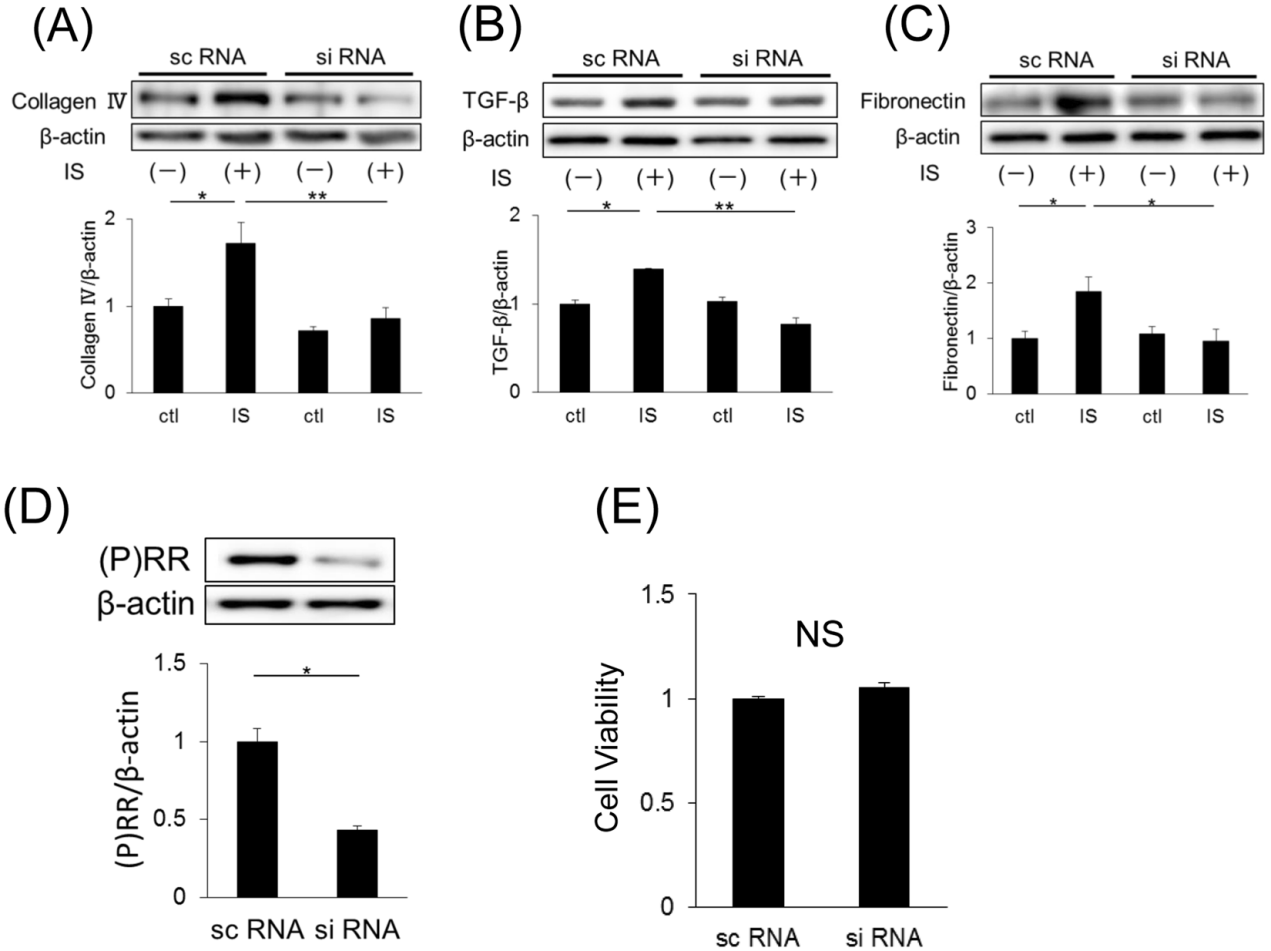
(**A**–**C**) Protein expression of type IV collagen (**A**), TGF-β (**B**) and fibronectin (**C**) in SV40 MES13 cells transfected with *(P)RR* small interfering RNA (siRNA) or nontargeting siRNA (scRNA) (n = 4 in each group). Cells were stimulated with 250 µM IS or 50 mM Tris-HCl (ctl) for 48 h. Mean ± SE (n = 4). *P < 0.05, **P < 0.01, by the Tukey–Kramer test. (**D**) (P)RR expression in SV40 MES13 cells transfected with *(P)RR* siRNA or scRNA. Mean ± SE (n = 4). *P < 0.05 compared with scRNA by the Student’s *t*-test. (**E**) Cell viability of SV40 MES13 cells treated with (*P)RR* siRNA or scRNA by the 3-(4,5-di-methylthiazol-2-yl)-2,5-diphenyltetrazolium bromide (MTT) assay (n = 6). Values are presented as the median (central line), interquartile range (box), and range (bars). NS, not significant, by the Student’s *t*-test.

As CKD progresses, accumulated IS induces the expression of fibrotic factors. The protein expression levels of type IV collagen, TGF-β and fibronectin significantly increased in SV40 MES13 cells after IS stimulation (Fig. 3A–C). To examine the role of (P)RR in IS-stimulated expression of these fibrotic factors, we knocked down (P)RR expression using small interfering RNA (siRNA). The greatest reduction in (P)RR expression (almost 50%) was observed 48 h after *(P)RR* siRNA transfection (Fig. 3D). Furthermore, (P)RR knockdown in SV40 MES13 cells did not affect cell growth or viability (Fig. 3E). Figure 3A–C also shows that IS did not increase the expression of the abovementioned fibrotic factors after (P)RR knockdown. These findings suggest that IS induced the expression of the fibrotic factors, collagen IV, TGF-β and fibronectin, through (P)RR in SV40 MES13 cells.

### Mesangial matrix remodeling by IS through (P)RR-mediated ERK1/2 pathway

**Figure 4:**
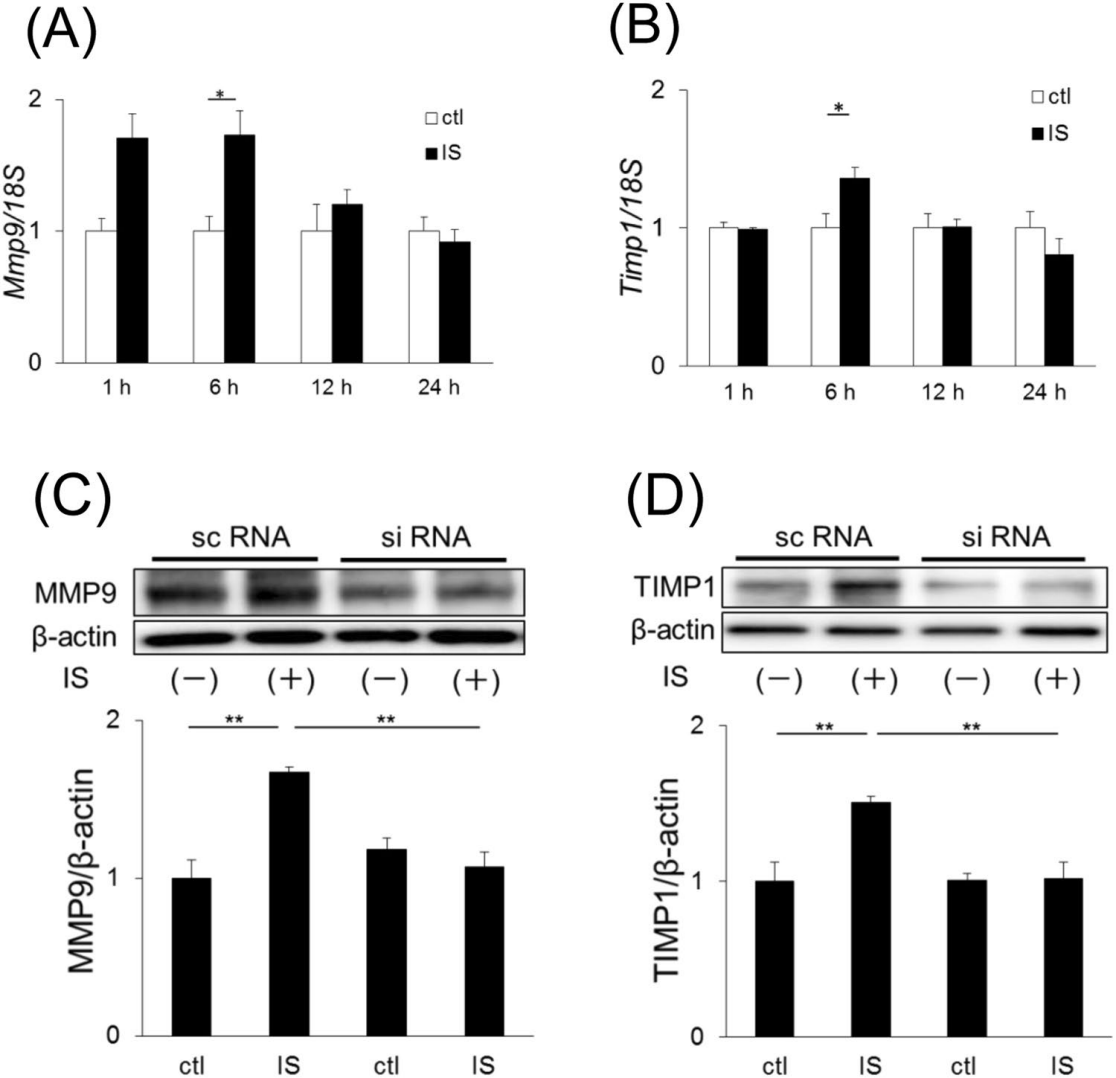
(**A** and **B**) mRNA expression of *Mmp9* (**A**) and *Timp1* (**B**) in SV40 MES13 cells. (**C** and **D**) Immunoblotting of MMP9 (**C**) and TIMP1 (**D**). Cells were stimulated with 250 µM IS or 50 mM Tris-HCl (ctl) for 48 h. Mean ± SE (n = 4). *P < 0.05, **P < 0.01, A and B were examined by the Student’s *t*-test. **C** and **D** were examined by the Tukey–Kramer test.

IS stimulation did not affect the cell proliferation of SV40 MES13 cells (Fig. 1E). We speculated that IS affects mesangial matrix expansion. To examine this speculation, we checked the expression of MMP9 and TIMP1 in SV40 MES13 cells. MMP9 and TIMP1 expression significantly increased after IS stimulation at both the mRNA (Fig. 4A, *P* = 0.005; 4B, *P* = 0.03) and protein levels (Fig. 4C, *P* = 0.008; 4D, *P* = 0.005), and the increases were inhibited by (P)RR knockdown (Fig. 4C and D).

**Figure 5:**
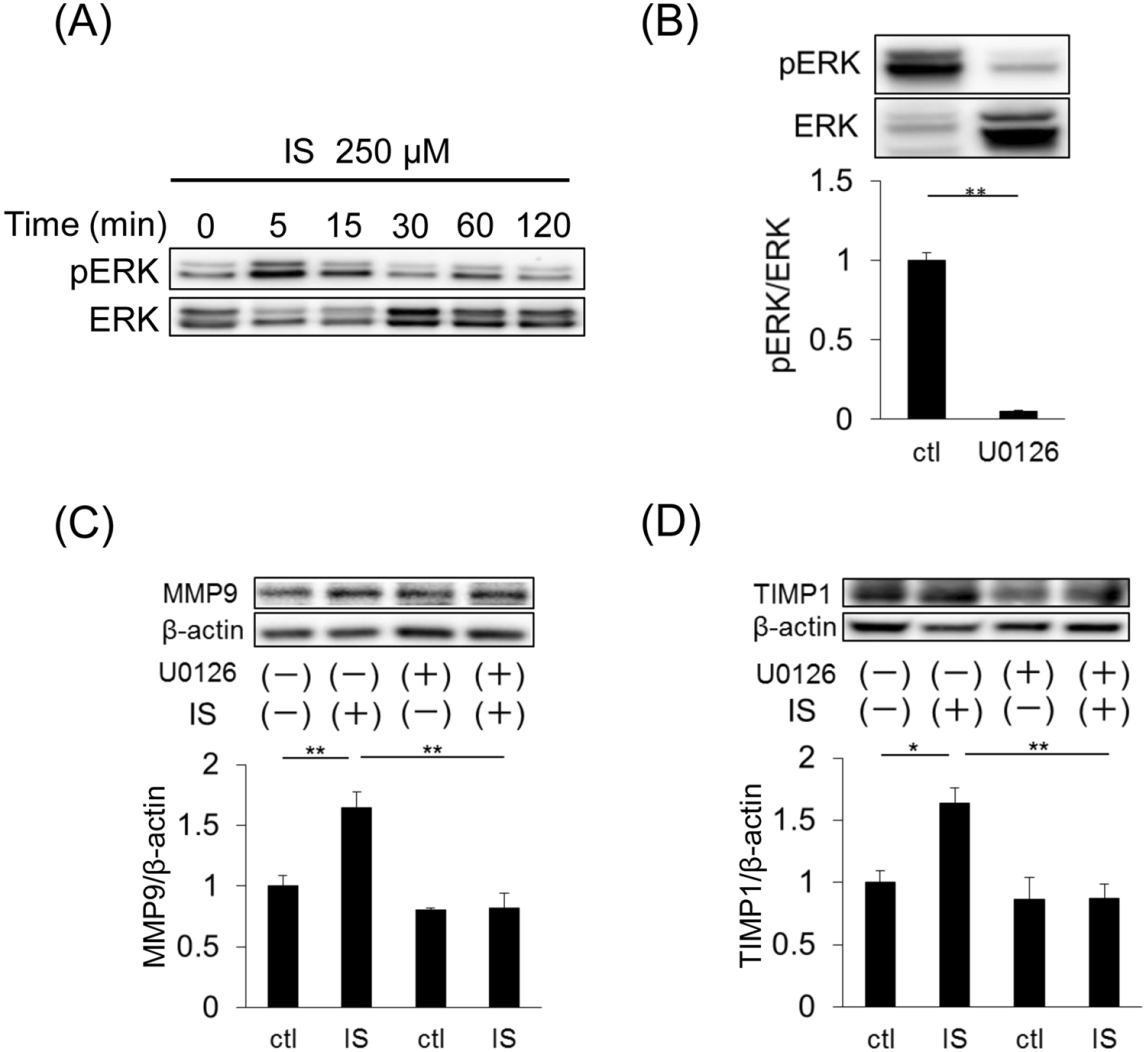
(**A**) Representative immunoblotting of phospho-ERK1/2 (pERK1/2) and total ERK1/2. (**B**) ERK1/2 phosphorylation by 250 µM IS stimulation was decreased in SV40 MES13 with U0126 (ERK1/2 specific inhibitor). Mean ± SE (n = 4). **P < 0.01, compared with control by the Student *t*-test. Cells were stimulated with 250 µM IS. (**C** and **D**) Immunoblotting of MMP9 (**C**) and TIMP1 (**D**). Cells were stimulated with 250 µM IS or 50 mM Tris-HCl (ctl) for 48 h after treatment with U0126. Mean ± SE (n = 4). *P < 0.05, **P < 0.01, by the Tukey–Kramer test.

IS induced ERK1/2 phosphorylation in SV40 MES13 cells (Fig. 5A). Next, we used the specific ERK1/2 inhibitor, U0126, to examine the effect of the ERK1/2 pathway on the induction of MMP9 and TIMP1. U0126 surely inhibited the ERK1/2 pathway (Fig. 5B). The increase in these proteins’ expression by IS stimulation was suppressed in the presence of U0126 (Fig. 5C and D). These results suggest that the expression of MMP9 and TIMP1 is induced by a (P)RR-mediated ERK1/2 pathway.

### Subject characteristics

The pathological diagnosis and characteristics of the subjects for CKD and IgA nephropathy(IgAN) patients at the time of biopsy are shown in Tables 1, 2 and 3. The renal biopsy specimens with a minimum of eight glomeruli were evaluated according to the Oxford Classification of IgAN (MEST score)^16^ by well-trained nephrologists (Supplementary Information). The index of glomerular lesion (IGL) (Supplementary Fig. 1) as an original index consists of mesangial cells and matrix, and is used for evaluation of the chronic phase in mesangial proliferative GN^17^ (Supplementary Information). In CKD patients, the mean age was 50.4 ± 19.1 years, the estimated glomerular filtration rate (eGFR) was 71.5 ± 30.8 mL/min/1.73 m^2^ and the urinary protein was 2.3 ± 3.5 g/gCr. On the other hands, in IgAN patients, the mean age was 47.0 ± 19.2 years, the eGFR was 77.3 ± 27.6 mL/min/1.73 m^2^, the urinary protein was 1.1 ± 2.1 g/gCr, IGL was 1.6 ± 0.4 and the interstitial fibrosis area was 22.4 ± 17.2%. Mesangial hypercellularity (M1) was present in 14.5%, segmental glomerulosclerosis (S1) was present in 60.0%, tubular atrophy/interstitial fibrosis (T1 + T2) was present in 21.8%, and endocapillary hypercellularity (E1) was present in 5.5% of the subjects.

**Table 1:** Subjects’ pathological diagnosis.

Pathological diagnosis	number
IgA nephropathy	55
Membranous nephropathy	9
Lupus nephritis	7
Diabetic glomerulosclerosis	5
Focal segmental glomerulosclerosis	5
Nephrosclerosis	4
Crescentic glomerulonephritis	4
Membranoproliferative glomerulonephritis	3
Tubulointerstitial nephritis	3
Endocapillary glomerulonephritis	2
Renal amyloidosis	2
Acute tubular necrosis	1
Total	100

**Table 2:** Subjects’ characteristics in CKD.

	patients (n = 100)
Age (years)	50.4 ± 19.1
Men (%)	55
BMI (kg/m^2^)	23.6 ± 4.0
DM (%)	14
Antihypertensive medication (%)	45
SBP (mmHg)	125.1 ± 18.2
DBP (mmHg)	74.3 ± 12.5
RAS inhibitor (%)	36
**Renal function, Urinalysis**	
Cr (mg/dL)	1.1 ± 1.2
eGFR (mL/min/1.73 m^2^)	71.5 ± 30.8
UP (g/gCr)	2.3 ± 3.5
**Laboratory data**	
s(P)RR (ng/mL)	13.9 ± 3.9
IS (μg/mL)	1.3 ± 1.4

Data are expressed as mean ± SD.

All parameters were measured at the renal biopsy.

BMI, body mass index; DM, diabetes mellitus; SBP, systolic blood pressure; DBP, diastolic blood pressure; RAS, renin angiotensin system; Cr, creatinine;

eGFR, estimated glomerular filtration rate; UP, urine protein.

**Table 3:** Subjects’ characteristics in IgA nephropathy.

	patients (n = 55)
Age (years)	47.0 ± 19.2
Men (%)	54.5
BMI (kg/m^2^)	23.3 ± 4.1
DM (%)	7.3
Antihypertensive medication (%)	38.2
SBP (mmHg)	122.5 ± 15.5
DBP (mmHg)	72.7 ± 11.2
RAS inhibitor (%)	39.4
**Renal function, Urinalysis**	
Cr (mg/dL)	0.88 ± 0.37
eGFR (mL/min/1.73 m^2^)	77.3 ± 27.6
UP (g/gCr)	1.1 ± 2.1
**Pathological indices**	
Oxford classification	
M1 (%)	14.5
E1 (%)	5.5
S1 (%)	60
T1 + T2 (%)*	21.8
**Laboratory data**	
s(P)RR (ng/mL)	13.55 ± 4.38
IS (μg/mL)	1.25 ± 0.97

Data are expressed as mean ± SD.

All parameters were measured at the renal biopsy.

BMI, body mass index; DM, diabetes mellitus; SBP, systolic blood pressure; DBP, diastolic blood pressure; RAS, renin angiotensin system; Cr, creatinine;

eGFR, estimated glomerular filtration rate; UP, urine protein;

M1, mesangial hypercellularity; E1, endocapillary hypercellularity; S1, segmental sclerosis; T1 + T2, tubular atrophy/interstitial fibrosis >25%;

IGL, index of glomerular lesion; s(P)RR, soluble (pro)renin receptor; IS, indoxyl sulfate.

*Because the number of T score positive subjects (T1: n = 7, T2: n = 5) was limited, we performed an association study according to the presence (T1 + T2) or absence (T0) of tubular atrophy/interstitial fibrosis.

### Correlation between s(P)RR and uremic toxins in CKD patients

There was a significant association between s(P)RR and IS (*P* = 0.02). However, there were not the correlations between s(P)RR and other uremic toxins (methylguanidine; *P* = 0.6, indole-3-acetic acid (IAA); *P* = 0.14) after adjustment of sex, age, BMI and diabetes.

### Correlation among s(P)RR and uremic toxins and renal histopathology in IgAN patients

We evaluated the correlation between s(P)RR and the clinical and pathological parameters.

**Figure 6:**
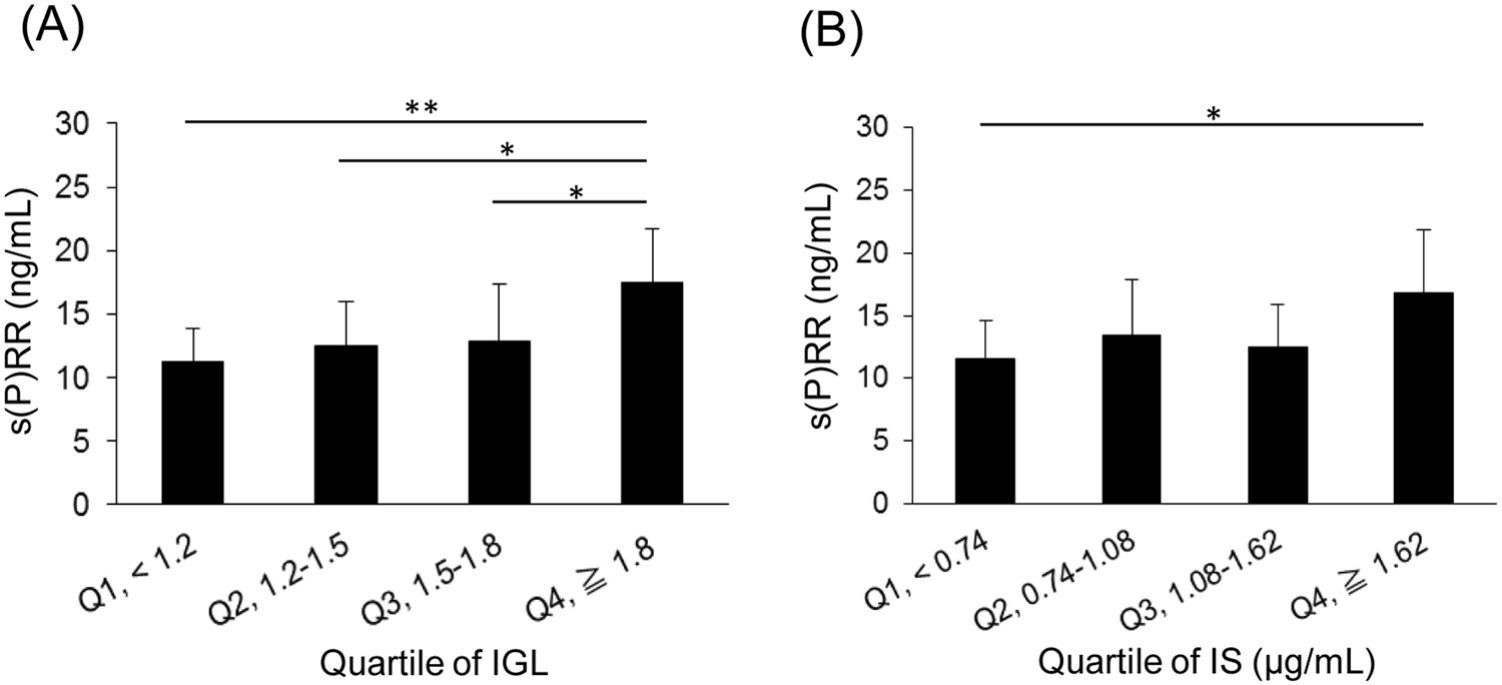
(**A**) Relationship between IGL levels and serum s(P)RR. Subjects were divided into four groups based on quartiles of IGL levels. The quartile ranges were: Q1, <1.2 (n = 14); Q2, 1.2–1.5 (n = 14); Q3, 1.5–1.8 (n = 13); Q4, ≥1.8 (n = 14). Mean ± SD (n = 55). The Kruscal-Wallis test was used (*P* = 0.001). *P < 0.05, **P < 0.01, by the the Steel–Dwass test. (**B**) Relationship between IS levels and serum s(P)RR. Subjects were divided into four groups based on quartiles of IS levels. The quartile ranges were: Q1, <0.74 (n = 14); Q2, 0.74–1.08 (n = 14); Q3, 1.08–1.62 (n = 14); Q4, ≥1.62 μg/mL (n = 13). Mean ± SD (n = 55). The Kruscal-Wallis test was used (*P* = 0.03). *P < 0.05, by the Steel–Dwass test.

The mean serum s(P)RR concentration was significantly higher in the presence of mesangial hypercellularity (*P* = 0.03) or segmental glomerulosclerosis (*P* = 0.004) in the MEST score (Table 4). Moreover, high IGL and IS levels were significantly associated with high concentrations of serum s(P)RR (Fig. 6). After adjustments for age, gender, body mass index and diabetes, the IGL (β = 0.41, *P* = 0.02) and IS levels (β = 0.12, *P* = 0.04) remained significantly associated with serum s(P)RR. In addition, we evaluate the correlation among s(P)RR, other uremic toxins and histopathology. Multivariate analysis (adjustment of sex, age, BMI and diabetes) revealed that there were the significant associations between s(P)RR and each uremic toxins (methylguanidine; *P* = 0.01, IAA; *P* = 0.001). However, there were not the correlation between IGL and them (methylguanidine; *P* = 0.68, IAA; *P* = 0.57).

**Table 4:** Correlations between s(P)RR and the MEST score in the Oxford Classification.

Histopathological change	s(P)RR (ng/mL)	*P* value	*P* value after adjustment
Mesangial hypercellularity			
M0 (n = 47)	13.06 ± 4.03		
M1 (n = 8)	16.46 ± 5.47	0.04	0.03
Endocapillary hypercellularity			
E0 (n = 52)	13.30 ± 4.20		
E1 (n = 3)	17.78 ± 6.48	0.09	0.21
Segmental glomerulosclerosis/adhesion			
S0 (n = 22)	11.55 ± 2.84		
S1 (n = 33)	14.89 ± 4.74	0.005	0.004
Tubular atrophy/Interstitial fibrosis			
T0 (n = 43)	12.92 ± 4.00		
T1 + T2 (n = 12)	15.83 ± 5.10	0.04	0.42

Data are expressed as mean ± SD.

Multivariable was adjusted for age, gender, body mass index and diabetes.

## Discussion

The present study revealed new aspects of (P)RR in the progression of mesangial fibrosis and matrix expansion. In this study, we showed that IS stimulated the expression of (P)RR. The expression of the fibrotic factors, TGF-β, type IV collagen and fibronectin, increased after IS stimulation, and these increases were significantly suppressed by (P)RR knockdown in SV40 MES13 cells. Although IS did not affect the number of mesangial cells, it significantly increased the expression of MMP9 and TIMP1 via (P)RR and the ERK1/2 pathway. In addition, the serum s(P)RR level correlated with IS in CKD patients. There was the association between s(P)RR and part of the MEST score in the Oxford Classification particularly in IgAN patients. Furthermore, higher IGL and serum IS levels were also associated with higher serum s(P)RR levels. These findings indicate that s(P)RR may be a biomarker of the degree of mesangial expansion and fibrosis, which would be helpful because it is difficult to predict chronic changes in glomeruli from laboratory data.

To confirm and extend our original observations that (P)RR activated by IS is involved in mesangial expansion, we performed experiments using the SV40 MES13 mouse mesangial cell line. We revealed that (P)RR expression was enhanced in SV40 MES13 cells in the presence of IS. In addition, IS induced the expression of the fibrotic factors, type IV collagen, TGF-β and fibronectin, and this induction was suppressed by (P)RR knockdown. Moreover, we demonstrated that IS affected mesangial matrix accumulation rather than cell proliferation. The deposition of extracellular matrix (ECM) is most commonly due to failures in the coordinated expression of proteins regulating collagen metabolism. In particular, an imbalance between MMPs and TIMPs is thought to be the main cause of increased matrix deposition^18^, consequently resulting in tissue fibrosis. MMP2 and MMP9 cleave collagen IV produced mainly by mesangial cells^19^, and TIMP1 and TIMP2 are strong inhibitors of MMPs. The expression of TIMP1 and MMP9 significantly increased by IS stimulation, which was suppressed by (P)RR knockdown in this study. In contrast, the mRNA expression of *TIMP2* and *MMP2* did not change after IS stimulation (data not shown). TIMPs were traditionally thought to control ECM proteolysis through direct inhibition of MMP-dependent ECM proteolysis. Thus, the upregulation of *TIMP1* mRNA may be a compensatory mechanism for the increase of MMP9 expression by IS stimulation. Furthermore, although TGF-β induces MMP9 secretion to digest excess ECM, chronic stimulation by TGF-β may become a maladaptation^20^. In CKD, chronic IS stimulation could induce maladaptive MMP9 through TGF-β, which may cause the matrix expansion/remodelling.

To evaluate active MMP9, we performed MMP9 activity assay. Although we used 10-fold concentrated culture medium of SV40 MES13, we could not detect both total and active MMP9. Thus, we tried the same experiment using human sera just before the introduction of hemodialysis. The result which total MMP9 was decreased was different from that of the present study. This difference is due to the exposure period. The patients are chronically exposed to uremic toxins, but the exposure time of IS was for 48 hours in our experiments. Indeed, it is reported that the production of MMP9 of patients on hemodialysis is decreased^21,22^.

It has been reported that RAS activation plays a role in mesangial expansion in IgAN model mice because administration of an angiotensin II type1 receptor blocker ameliorated the extent of mesangial matrix expansion^23^. The inhibition of (P)RR or ERK1/2 signalling attenuated mesangial expansion and decreased the expression of fibrotic factors^24,25^. Indeed, we found that the (P)RR and ERK1/2 pathway was involved in the induction of MMP9 and TIMP1. Furthermore, the binding of renin or prorenin to (P)RR activates the ERK1/2 signalling^1^. Thus, the (P)RR-mediated ERK1/2 pathway may be associated with mesangial expansion based on the increase in the expression of TIMP1 and MMP9.

We identified that there was a significant association between s(P)RR and IS in CKD patient. Moreover, we evaluated the correlation among s(P)RR, uremic toxins and histopathological change in IgAN patients because IgAN is characterized by mesangial expansion. Although s(P)RR correlated with IGL and IS significantly, there was not significant association between IGL and other uremic toxins. Actually, western blot analysis revealed that (P)RR was not increased by methylguanidine and hippuric acid in SV40 MES13. Therefore, uremic toxins which increased (P)RR expression or have the significant association with IGL could be limited. As there are as yet no reliable biomarkers for mesangial expansion, s(P)RR may be a promising marker which we could predict the level of mesangial expansion at diagnosis or follow up period.

This is the first study reporting an association of s(P)RR with mesangial hypercellularity (M1) and segmental glomerulosclerosis (S1) in the MEST score. In contrast, tubular atrophy/interstitial fibrosis (T1) and extracapillary hypercellularity (E1) showed no significant association. Mesangial matrix accumulation has been reported to result in glomerulosclerosis^26^. Furthermore, we demonstrated that the serum s(P)RR concentration was independently associated with IGL. IGL is a predictor of mesangial proliferation and sclerotic change^27–29^. These results suggest that (P)RR is involved in mesangial injury. In addition, the serum IS concentration was independently related to the s(P)RR concentration in IgAN patients. Previous studies have indicated that IS is involved in glomerulosclerosis^9,10^, and that it upregulates prorenin expression in proximal tubular cells^30^. Taken together, our pathological analysis of IgAN patients suggests that (P)RR contributes to mesangial injury in the presence of IS.

As a limitation, we used mouse mesangial cell line *in vitro* study. However, only humans and higher primates have IgA1 subclass, of which –glycosylation is linked to the pathogenesis of IgAN. Further investigation in human mesangial cells will help to elucidate the role of (P)RR in IgAN.

In conclusion, (P)RR is involved in mesangial fibrosis and matrix expansion through ERK1/2 phosphorylation in the presence of IS. Clinically, s(P)RR may be used as a biomarker of mesangial fibrosis and matrix expansion.

## Materials and Methods

### Cell culture

SV40 MES13, a mouse mesangial cell line, was obtained from American Type Culture Collection (Manassas, VA) and was cultured in DMEM/F12 medium (Wako, Tokyo, Japan) with 14 mM HEPES (Nacalai tesque, Kyoto, Japan), 5% fetal bovine serum (Life Technologies, Grand Island, NY), 100 U/mL penicillin and 100 µg/mL streptomycin (Sigma-Aldrich, Munich, Germany). Cells were incubated in a humidified incubator with 5% CO_2_ at 37 °C. Cells were serum-starved for 24 h before IS stimulation experiments. We used 50 mM Tris-HCl buffer as a vehicle control because of the IS solvent.

(P)RR siRNA (L-063641-01-0005, Thermo Scientific, Waltham, MA) or non-targeting control siRNA (D-001810-10-20, Thermo Scientific) were transfected into SV40 MES13 cells (final concentration, 10 nM) by Lipofectamin2000 (Thermo Scientific) according to the manufacturer’s protocol.

To assess the effects of IS or siRNA on mesangial cell growth and viability, the 3-(4,5-di-methylthiazol-2-yl)-2,5-diphenyltetrazolium bromide (MTT) assay (10009365; Cayman Chemical Co., Ann Arbor, MI) was performed according to the manufacturer’s protocol.

### Immunohistochemistry

Slides were prepared with SV40 MES13 cells stimulated by IS for 48 h, followed by incubation with fixation buffer (00-8222-49, eBioscience, San Diego, CA) for 30 min. Slides were incubated overnight at 4 °C with primary antibodies [anti-(P)RR (homemade) or normal rabbit serum (as a control); 1:5000] in protein block serum-free solution (X0909, Dako, Glostrup, Denmark). Antiserum against (P)RR was raised in a rabbit by injecting a human (P)RR fragment corresponding to amino acids 224–237 conjugated to BSA^31,32^. After washing, slides were incubated with Alexa Fluor 555 goat anti-rabbit antibody (A-21428, Life technologies; 1:1000) for 30 min in protein block serum-free solution. Then, slides were embedded in DAPI-Fluoromount-G (SouthernBiotech, Birmingham, AL) and observed by C2Si confocal microscopy (Nikon, Tokyo, Japan).

### Real-time quantitative polymerase chain reaction analysis

Total RNA was extracted by RNeasy Mini Kit (Qiagen, Germantown, MD), and was reverse transcribed with SuperScriptIII (Invitrogen, Carlsbad, CA). Five nanograms of cDNA were amplified in duplicate using SYBR Premix Ex Taq (TaKaRa, Otsu, Japan) and CFX96 (Bio-Rad, Hercules, CA). Primers for *Mmp9* (GenBank accession no. NM013599) and *Timp1* (NM001044384) were purchased from TaKaRa Bio. Primers for *18* *S* rRNA (NR003278) were designed by Life Technologies: sense 5′-GTAACCCGTTGAACCCCATT-3′ and anti-sense 5′-CCATCCAATCGGTAGTAGCG-3′. After heating at 95 °C for 2 min, 39 cycles of denaturation, annealing and elongation were carried out at 95 °C for 5 s, 60 °C for 20 s and, 65 °C for 5 s, respectively. The mRNA expression levels were normalized to *18* *S* rRNA.

### Western blotting

SV40 MES13 cells suspended with RIPA buffer (Cell Signaling Technology, Danvers, MA) containing a protease inhibitor cocktail (Roche, Basel, Switzerland) were centrifuged at 17000 × *g* for 10 min and the supernatant was resuspended with Laemmli Sample Buffer (161-0737, Bio-Rad) and β-mercaptoethanol (Bio-Rad). Protein extracts were separated by SDS-PAGE for 90 min at 150 V and transferred to polyvinylidene difluoride (PVDF) membranes (Bio-Rad) by a Trans-Blot (Bio-Rad). Membranes were incubated in PVDF blocking buffer (TOYOBO, Osaka, Japan) for 1 h at room temperature (RT), and then with primary antibodies [anti-(P)RR, 1:5000, homemade; anti-TGF-β1, 1:1000, sc-146, Santa Cruz Biotechnology, Santa Cruz, CA; anti-Collagen IV, 1:1000, ab6586, abcam; anti-Fibronectin, 1:1000, F3648, Sigma-Aldrich; anti-TIMP1, 1:1000, ab38978, abcam; anti-MMP9, 1:1000, ab38898, abcam; anti-ERK1/2, 1:1000, 4695S, Cell Signaling Technology; anti-p-ERK1/2, 1:1000, 4377S, Cell Signaling Technology] in Can get signal Solution 1 (TOYOBO) overnight at 4 °C. Membranes were washed three times with Tris-buffered saline (TBS) containing 0.08% Tween 20 and incubated with horseradish peroxidase (HRP)-conjugated secondary antibodies (anti-rabbit IgG, 1:1000, sc-2004; anti-mouse IgG, 1:5000, sc-2005; Santa Cruz Biotechnology) in Can get signal Solution 2 (TOYOBO) for 1 h at RT. After washing three times with TBS containing 0.08% Tween 20, chemiluminescence (ECL Western blotting detection system; Amersham, Arlington Heights, IL) was measured using VersaDocMP5000 (Bio-Rad). β-actin expression (1:5000, sc-47778, Santa Cruz Biotechnology) was used as an internal control.

### ERK1/2 phosphorylation upon IS stimulation

To examine whether IS stimulation induces the expression of MMP9 and TIMP1 through the ERK1/2 pathway, cells were incubated for 60 min with the specific ERK1/2 inhibitor, U0126 (10 μmol/L, U120, Sigma-Aldrich), in DMEM containing penicillin G (100 IU/mL) and streptomycin (100 µg/mL). Thereafter, the cells were incubated in medium containing 250 µM IS for 48 h.

### Patients and sample collection

Data and serum samples were collected from 100 patients performed renal biopsy between 2014 and 2016 at the Tohoku University Hospital. Sera were obtained from the peripheral blood on the day of the renal biopsy and stored at −80 °C until use. The study was performed under the declaration of Helsinki principles. The study protocol was approved by the Institutional Review Board of the Tohoku University School of Medicine (registration number: 2010-108). All participants provided written informed consent after a full explanation of the purpose of the study and the potential risk involved.

### Clinical evaluation

The clinical and laboratory data of these patients on the day of the renal biopsy were retrospectively analysed. Diabetes was defined as random blood glucose ≥11.1 mmol/L (≥200 mg/dL), HbA1c ≥6.5% according to the Japan Diabetes Society, using medication to control diabetes, and/or a history of diabetes. The GFR was calculated using the new Japanese equation^33^. Uremic toxins including IS, methylguanidine and IAA were measured by liquid chromatography–mass spectrometry (LC-MS)/MS as previously reported^34^. Serum s(P)RR levels were measured using an enzyme-linked immunosorbent assay (ELISA) kit (Immuno-Biological Laboratories Co., Tokyo, Japan), consisting of a solid-phase sandwich ELISA with two kinds of highly specific antibodies^35^.

### Statistical analysis

Values are expressed as the mean ± standard error (SE). Data were analysed using JMP software version 12 (SAS Institute Inc., Cary, NC). Multiple groups were compared using ANOVA with the Tukey–Kramer test for parametric values. Otherwise, the Kruscal–Wallis test, followed by the Steel–Dwass test was used for non-parametric values. *P* < 0.05 was considered statistically significant.

### Data availability

All data generated or analysed during this study are included in this published article and its Supplementary Information files.

## Electronic supplementary material

Supplementary Information

## References

[1] Nguyen G (2002). Pivotal role of the renin/prorenin receptor in angiotensin II production and cellular responses to renin. J Clin Invest..

[2] Cousin C (2009). Soluble form of the (pro)renin receptor generated by intracellular cleavage by furin is secreted in plasma. Hypertension..

[3] Gonzalez AA, Lara LS, Luffman C, Seth DM, Prieto MC (2011). Soluble form of the (pro)renin receptor is augmented in the collecting duct and urine of chronic angiotensin II-dependent hypertensive rats. Hypertension..

[4] Huang Y (2006). Renin increases mesangial cell transforming growth factor-beta1 and matrix proteins through receptor-mediated, angiotensin II-independent mechanisms. Kidney Int..

[5] Ichihara A, Kaneshiro Y, Takemitsu T, Sakoda M, Itoh H (2007). The (pro)renin receptor and the kidney. Seminars in nephrology..

[6] Narumi K (2015). A functional (pro)renin receptor is expressed in human lymphocytes and monocytes. Am J Physiol Renal Physiol..

[7] Kaneshiro Y (2007). Slowly progressive, angiotensin II-independent glomerulosclerosis in human (pro)renin receptor-transgenic rats. J Am Soc Nephrol..

[8] Hamada K (2013). Serum level of soluble (pro)renin receptor is modulated in chronic kidney disease. Clin Exp Nephrol..

[9] Niwa T, Ise M (1994). Indoxyl sulfate, a circulating uremic toxin, stimulates the progression of glomerular sclerosis. J Lab Clin Med..

[10] Niwa T, Ise M, Miyazaki T (1994). Progression of glomerular sclerosis in experimental uremic rats by administration of indole, a precursor of indoxyl sulfate. Am J Nephrol..

[11] Li S (2016). Indoxyl Sulfate Induces Mesangial Cell Proliferation via the Induction of COX-2. Mediators of inflammation..

[12] Miyazaki T, Ise M, Seo H, Niwa T (1997). Indoxyl sulfate increases the gene expressions of TGF-beta 1, TIMP-1 and pro-alpha 1(I) collagen in uremic rat kidneys. Kidney Int Suppl..

[13] Yisireyili M (2014). Indoxyl sulfate-induced activation of (pro)renin receptor promotes cell proliferation and tissue factor expression in vascular smooth muscle cells. PLoS One..

[14] Saito S (2014). Indoxyl sulfate-induced activation of (pro)renin receptor is involved in expression of TGF-beta1 and alpha-smooth muscle actin in proximal tubular cells. Endocrinology..

[15] Vanholder R (2003). Review on uremic toxins: classification, concentration, and interindividual variability. Kidney Int..

[16] Working Group of the International Ig, A. N. N. *et al*. The Oxford classification of IgA nephropathy: rationale, clinicopathological correlations, and classification. *Kidney Int*. **76**, 534–545 (2009). 10.1038/ki.2009.243 19571791

[17] Suwa N, T. T. Morphological and *Morphometrical Analy*sis of Circulation in Hypertension and Ischemic Kidney. Munchen-Berlin-Wien, Germany, Urban & Schwarzenberg, pp 108–116 (1971).

[18] Woessner JF (1991). Matrix metalloproteinases and their inhibitors in connective tissue remodeling. FASEB J..

[19] Martin J, Eynstone L, Davies M, Steadman R (2001). Induction of metalloproteinases by glomerular mesangial cells stimulated by proteins of the extracellular matrix. J Am Soc Nephrol..

[20] Li SY (2014). Matrix metalloproteinase-9 deficiency attenuates diabetic nephropathy by modulation of podocyte functions and dedifferentiation. Kidney Int..

[21] Pawlak K, Mysliwiec M, Pawlak D (2011). Peripheral blood level alterations of MMP-2 and MMP-9 in patients with chronic kidney disease on conservative treatment and on hemodialysis. Clinical biochemistry..

[22] Rysz J (2011). Serum metalloproteinases MMP-2, MMP-9 and metalloproteinase tissue inhibitors TIMP-1 and TIMP-2 in patients on hemodialysis. International urology and nephrology..

[23] Ohashi N (2009). Role of activated intrarenal reactive oxygen species and renin-angiotensin system in IgA nephropathy model mice. Clin Exp Pharmacol Physiol..

[24] He M (2009). Inhibition of renin/prorenin receptor attenuated mesangial cell proliferation and reduced associated fibrotic factor release. Eur J Pharmacol..

[25] Li XQ (2016). Corosolic acid inhibits the proliferation of glomerular mesangial cells and protects against diabetic renal damage. Sci Rep..

[26] Kashgarian M, Sterzel RB (1992). The pathobiology of the mesangium. Kidney Int..

[27] Hotta O (1993). Predictive value of small crescents in IgA nephropathy: analysis of four patients showing a deteriorated renal function during a long follow-up period. Clin Nephrol..

[28] Soma J (1995). Participation of CR1 (CD35), CR3 (CD11b/CD18) and CR4 (CD11c/CD18) in membranoproliferative glomerulonephritis type I. Clinical and experimental immunology..

[29] Hotta O, Furuta T, Chiba S, Tomioka S, Taguma Y (2002). Regression of IgA nephropathy: a repeat biopsy study. Am J Kidney Dis..

[30] Saito S, Yisireyili M, Shimizu H, Ng HY, Niwa T (2015). Indoxyl sulfate upregulates prorenin expression via nuclear factor-kappaBp65, signal transducer and activator of transcription 3, and reactive oxygen species in proximal tubular cells. J Ren Nutr..

[31] Hirose T (2009). Association of (pro)renin receptor gene polymorphism with blood pressure in Japanese men: the Ohasama study. Am J Hypertens..

[32] Hirose T (2011). Association of (pro)renin receptor gene polymorphisms with lacunar infarction and left ventricular hypertrophy in Japanese women: the Ohasama study. Hypertens Res..

[33] Imai E (2007). Estimation of glomerular filtration rate by the MDRD study equation modified for Japanese patients with chronic kidney disease. Clin Exp Nephrol..

[34] Sato E (2016). Metabolic alterations by indoxyl sulfate in skeletal muscle induce uremic sarcopenia in chronic kidney disease. Sci Rep..

[35] Maruyama N, Segawa T, Kinoshita N, Ichihara A (2013). Novel sandwich ELISA for detecting the human soluble (pro)renin receptor. Front Biosci (Elite Ed)..

