# Supplementary material for: (Pro)renin receptor is involved in mesangial fibrosis and matrix expansion
Source: Sci Rep. 2018 Jan 8;8:16. doi: 10.1038/s41598-017-18314-w (PMC5758707; doi:10.1038/s41598-017-18314-w)
Supplement: Supplementary file 1 — Supplementary Information [file 41598_2017_18314_MOESM1_ESM.pdf]

(Pro)renin receptor is involved in mesangial fibrosis and matrix expansion

Kaori Narumi [1], Emiko Sato [1,2], Takuo Hirose [1], Tae Yamamoto [1], Takashi Nakamichi [1], Mariko Miyazaki [1], Hiroshi Sato [1,2], Sadayoshi Ito [1]

[1]Division of Nephrology, Endocrinology and Vascular Medicine, Tohoku University Graduate School of Medicine, 1-1 Seiryo-machi, Aoba-ku, Sendai 980-8574, Japan.
[2]Division of Clinical Pharmacology and Therapeutics, Tohoku University Graduate School of Pharmaceutical Science, 6-3 Aoba, Aramaki, Aoba-ku, Sendai 980-8579, Japan.

Running head: (P)RR in mesangial cells

Correspondence to: Kaori Narumi
Division of Nephrology, Endocrinology, and Vascular Medicine, Tohoku University Graduate School of Medicine, 1-1 Seiryo-cho, Aoba-ku, Sendai 980-8574, Japan.
Tel: +81-22-717-7163; Fax: +81-22-717-7168
k-n-0922@med.tohoku.ac.jp

Supplementary Figure 1

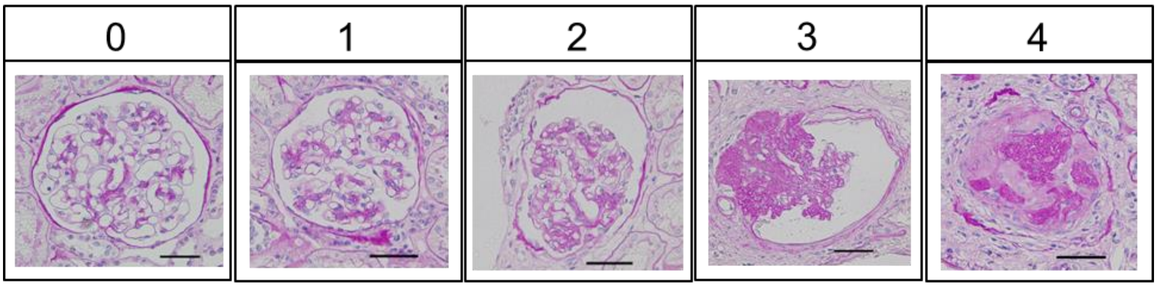

$$\mathrm{IGL} = \frac{(0\times n_0)+(1\times n_1)+(2\times n_2)+(3\times n_3)+(4\times n_4)}{n_0+n_1+n_2+n_3+n_4}$$

IGL calculation. Representative glomerulus of each grade with periodic acid-Schiff staining. The degree of morphological damage in each glomerulus was graded from 0 to 4 according to the percentage of mesangial proliferation and sclerosis. The average of all glomeruli was calculated as IGL. Scale bars, 50 µm.

n_0–n_4, the number of glomeruli showing changes of grade 0 to 4.

Supplementary Methods

The renal biopsy specimens were evaluated according to the Oxford Classification of IgA nephropathy (MEST score)[1] by well-trained nephrologists. The MEST score includes the following four histological components: mesangial hypercellularity mesangial score ≤ 0.5 (M0), > 0.5, or > 50% of glomeruli with ≥ 4 mesangial cells per mesangial area (M1), endocapillary hypercellularity absent (E0) or present (E1), segmental glomerulosclerosis absent (S0) or present (S1), tubular atrophy/interstitial fibrosis < 25% (T0), 25–50% (T1), > 50% (T2). The index of glomerular lesion (IGL) as an original index consists of mesangial cells and matrix, and is used for evaluation of the chronic phase in mesangial proliferative glomerulonephritis [2]; it is converted into numerical values by the degree of sclerotic changes as previously described (score 0–4; 0: no glomerular change; 1: lesion localized to mesangial area; 2: focal structure collapse of capillary; 3: remarkable structure collapse of capillary; 4: global sclerosis) (**Supplementary Fig. 1**) [3-6]. IGL has been reported to be a predictor of renal function and of the response to treatment in IgA nephropathy patients [4]. The interstitial fibrosis area was determined with the Verhoeff and Masson trichrome stain.

Supplementary References

1 Working Group of the International Ig, A. N. N. *et al.* The Oxford classification of IgA nephropathy: rationale, clinicopathological correlations, and classification. *Kidney Int.* **76**, 534-545 (2009).

2 Suwa N, T. T. Morphological and Morphometrical Analysis of Circulation in Hypertension and Ischemic Kidney. *Munchen-Berlin-Wien, Germany, Urban & Schwarzenberg*, pp 108-116 (1971).

3 Sato, Y., Hotta, O., Taguma, Y., Takasaka, T. & Nose, M. IgA nephropathy with poorly developed lymphoepithelial symbiosis of the palatine tonsils. *Nephron*. **74**, 301-308 (1996).

4 Hotta, O. *et al.* Predictive value of small crescents in IgA nephropathy: analysis of four patients showing a deteriorated renal function during a long follow-up period. *Clin Nephrol*. **40**, 125-130 (1993).

5 Ootaka, T. *et al.* Histologic localization of terminal complement complexes in renal diseases. An immunohistochemical study. *Am J Clin Pathol*. **91**, 144-151 (1989).

6 Hotta, O. *et al.* Tonsillectomy and steroid pulse therapy significantly impact on clinical remission in patients with IgA nephropathy. *Am J Kidney Dis*. **38**, 736-743 (2001).

Supplementary Figure 2

(a)

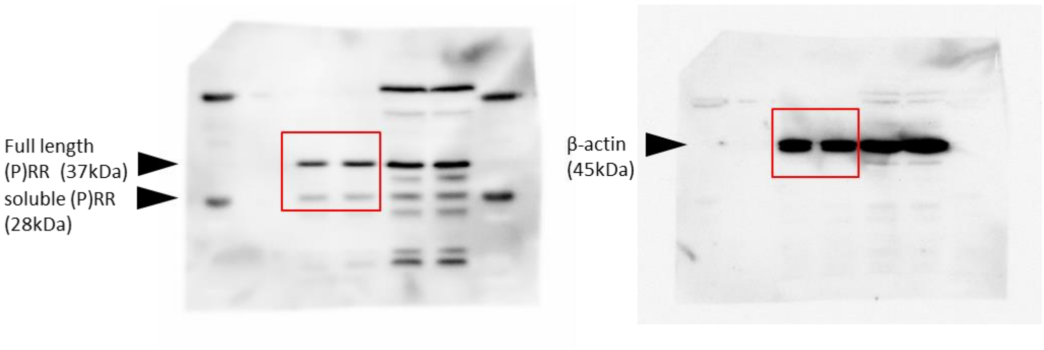

(b)

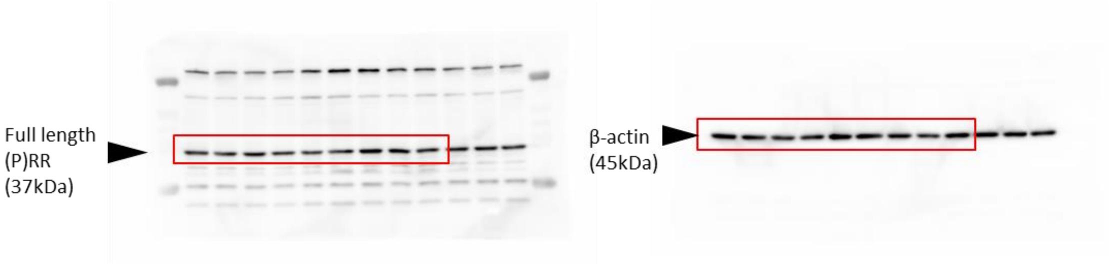

(c)

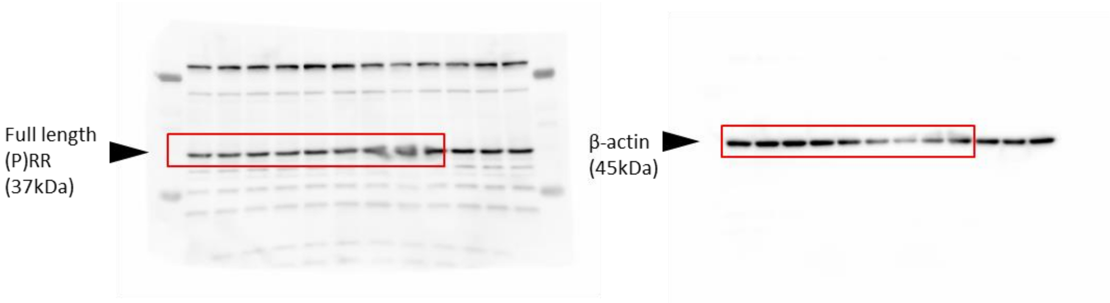

Supplementary Figure 2. (a) (P)RR protein expression in SV40 MES13 cells assessed by western blot analysis. (b and c) SV40 MES13 cells were incubated with the indicated concentration of IS for 24 h (b) or 48 h (c). Highlighted lanes of unedited gel correspond to Figure 2.

Supplementary Figure 3

(a)

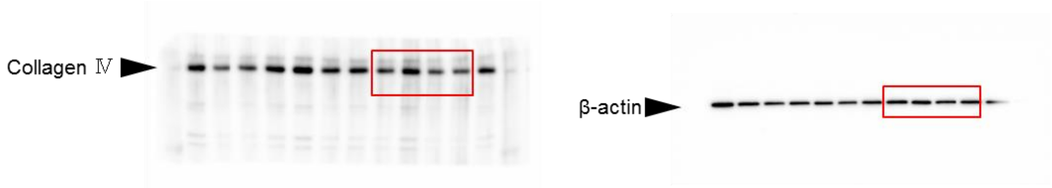

(b)

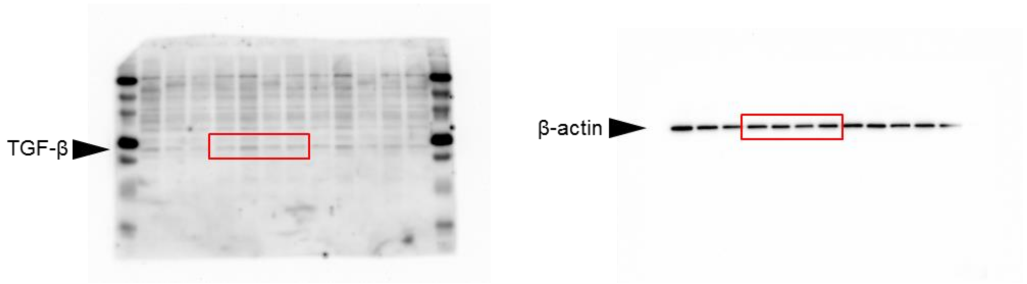

(c)

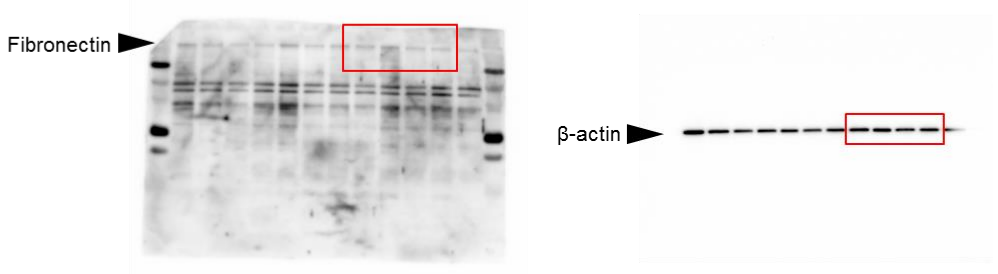

(d)

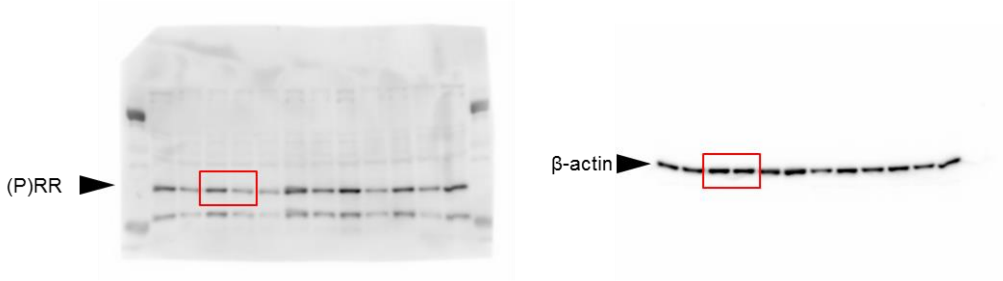

Supplementary Figure 3. (a-c) Immunoblotting of type IV collagen (a), TGF-β (b) and fibronectin (c) in SV40 MES13 cells transfected with (P)RR siRNA or scRNA. Cells were stimulated with 250 µM IS or 50mM Tris-HCl (ctl) for 48 h. (d) (P)RR expression in SV40 MES13 cells transfected with (P)RR siRNA or scRNA. Highlighted lanes of unedited gel correspond to Figure 4.

Supplementary Figure 4

(a)

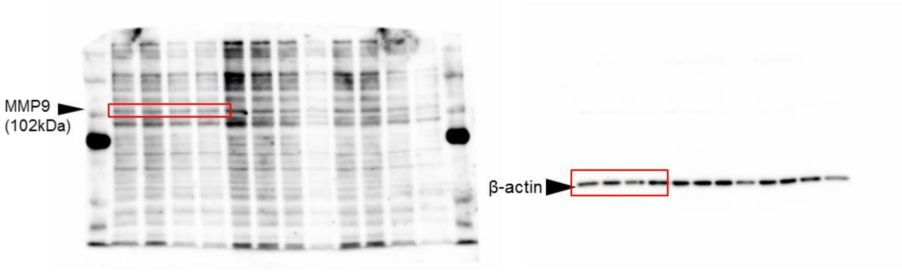

(b)

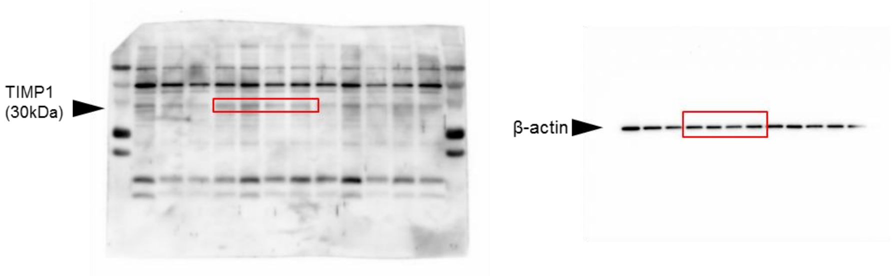

Supplementary Figure 4. (a and b) Immunoblotting of MMP9 (a) and TIMP1 (b). Cells were stimulated with 250 µM IS or 50mM Tris-HCl (ctl) for 48 h. Highlighted lanes of unedited gel correspond to Figure 5.

Supplementary Figure 5

(a)

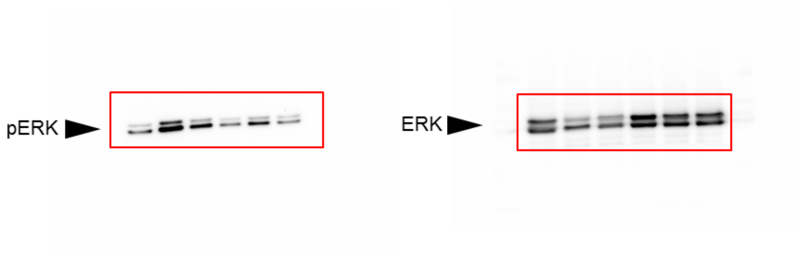

(b)

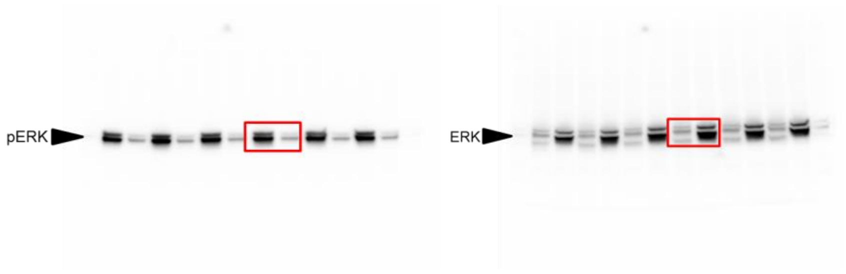

(c)

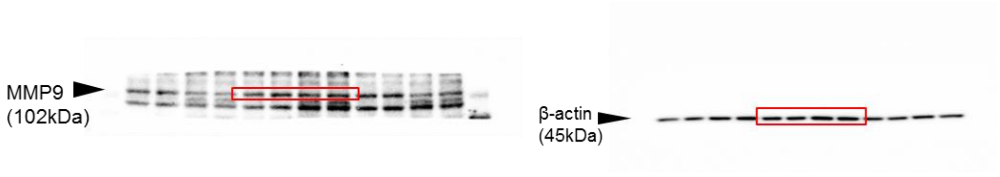

(d)

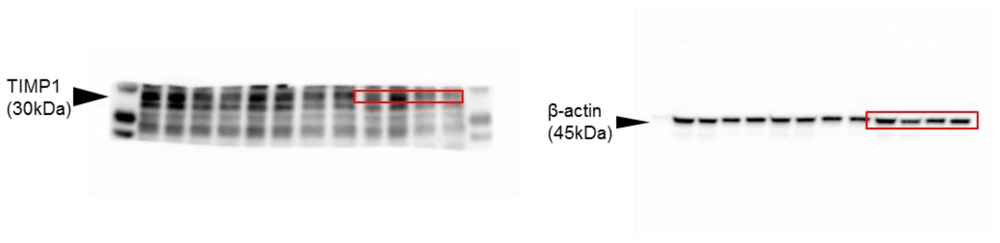

Supplementary Figure 5. (a) Immunoblotting of phospho-ERK1/2 (pERK1/2) and total ERK1/2. Cells were stimulated with 250 µM IS. (b) Immunoblotting of phosphor-ERK1/2 (pERK1/2) and total ERK1/2. Cells were stimulated by 250 µM IS in the medium with or without U0126 (ERK1/2 specific inhibitor). (c and d) Immunoblotting of MMP9 (c) and TIMP1 (d). Cells were stimulated with 250 µM IS or 50 mM Tris-HCl (ctl) for 48 h after treatment of U0126. Highlighted lanes of unedited gel correspond to Figure 6.

Supplementary Figure 6

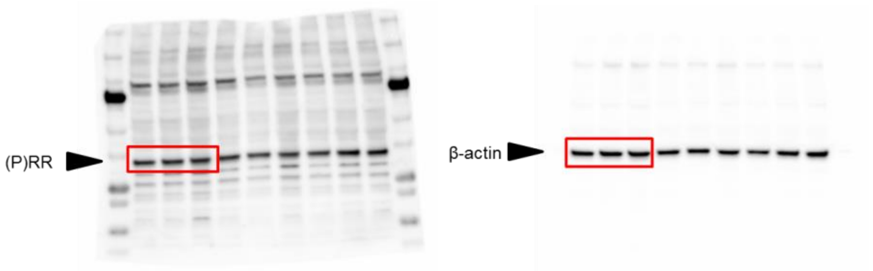

Supplementary Figure 6. Immunoblotting of (P)RR. Cells were stimulated with 50 mM Tris-HCl or other uremic toxins including methylguanidine and hippuric acid. Highlighted lanes of unedited gel correspond to the result of (P)RR expression to Reviewer 2.
